# Facile Synthesis of Bioactive Silver Nanocomposite Hydrogels with Electro-Conductive and Wound-Healing Properties

**DOI:** 10.3390/gels11020084

**Published:** 2025-01-22

**Authors:** Solaiman, Tahmina Foyez, Syed Abdul Monim, Aminur Rahman, Abu Bin Imran

**Affiliations:** 1 Department of Chemistry, Bangladesh University of Engineering and Technology, Dhaka 1000, Bangladesh; solaiman.chem@gmail.com (S.); 0417034005@chem.buet.ac.bd (S.A.M.); aminurbncd@gmail.com (A.R.); 2 Department of Pharmacy, United International University, United City, Madani Ave, Dhaka 1212, Bangladesh

**Keywords:** electro-conductive, bioactive, wound healing, silver nanoparticles, nanocomposite, hydrogels

## Abstract

Bioactive metal and metal oxide-based nanocomposite hydrogels exhibit significant antibacterial properties by interacting with microbial DNA and preventing bacterial replication. They offer potential applications as coating materials for human or animal skin injuries to prevent microbial growth and promote healing. In this study, silver nanoparticles (AgNPs) were synthesized using a chemical reduction method and incorporated into a polymer network to fabricate silver nanocomposite hydrogels (AgNCHGs) through a simple free radical polymerization method. *N*-isopropylacrylamide (NIPA), which has lower critical solution temperature (LCST) at about body temperature, or acrylamide (AAm) was used as the main monomer, while one or more ionic co-monomers, such as acrylic acid (AAc) and 2-acrylamido-2-methylpropane sulfonic acid (AMPS), were incorporated to obtain AgNCHGs. AgNPs were introduced into the hydrogel network via three different approaches. In the first method, the synthesized hydrogel was immersed in a silver nitrate (AgNO_3_) solution and reduced in situ using sodium borohydride (NaBH_4_) as a reducing agent. The second method involved mixing AgNO_3_ with gel precursors before reduction with NaBH_4_ to form AgNPs within the hydrogel. The final approach synthesized the AgNCHGs directly in a dispersion of pre-fabricated AgNPs. The incorporation of AgNPs in different AgNCHGs was confirmed through various characterization techniques. Varying temperature and pH conditions can trigger the release of bioactive AgNPs from the hydrogels. Furthermore, the antimicrobial and wound-healing properties of the AgNCHGs were evaluated against bacteria and fungi, demonstrating their potential in biomedical applications. In addition, AgNCHGs exhibit excellent electrical conductivity. The electrical conductivity of the hydrogels can be finely tuned by adjusting the concentration of AgNPs, making these materials promising candidates for energy, sensor, and stretchable electronics applications. This study presents facile synthesis methods of AgNCHGs, which integrate bioactivity, wound healing, and electrical conductivity in the same matrix, addressing a significant challenge in designing multifunctional hydrogels for next-generation technologies.

## 1. Introduction

The design and development of nanocomposite and nanostructured materials have opened up a new era for constructing a well-designed novel class of materials for catalytic, optical, electronic, and biomedical applications [1,2]. Nanoparticles possess a high surface area-to-volume ratio, enhancing their antimicrobial efficacy against bacteria and fungi. Metals and metal oxides at the nanoscale, such as silver, sulfur, and zinc oxide, have demonstrated effectiveness against various pathogenic microorganisms [3,4,5,6]. They exhibit strong antimicrobial properties by attaching to microbial DNA and inhibiting the process of bacterial replication [7]. However, often, they cannot be used directly in an uncontrolled manner due to their toxicity.

AgNPs exhibit outstanding optical characteristics and have a remarkable ability to enhance signals in both fluorescence and Raman spectroscopies [8,9]. Their chemical inertness and straightforward synthesis have led to diverse scientific and practical applications, particularly in healthcare, electronics, and environmental sectors. They are extensively used in wound dressings, drug delivery systems, biosensors, and antimicrobial coatings for medical devices and textiles [10,11,12,13,14]. They are also integrated into water purification systems and food packaging to prevent contamination while serving as catalysts in various chemical reactions. Additionally, AgNPs are included in personal care products due to their preservative and antimicrobial effects. However, one of the significant challenges in applying AgNPs is their propensity for aggregation, which can severely compromise their stability and functionality. When AgNPs aggregate, their effective surface area diminishes, leading to reduced antimicrobial and catalytic activity and alterations in their optical and electrical properties. This aggregation also affects the uniformity and distribution of nanoparticles across different matrices, resulting in inconsistent performance in applications such as drug delivery, coatings, and electronics. Despite the challenges posed by aggregation, AgNPs continue to be a focus of extensive research and development, particularly in improving their stability, controlling particle size, and preventing oxidation. While stabilizers or capping agents are commonly employed to enhance the performance of AgNPs, identifying the most effective solution for each specific application remains a complex task. Soft and versatile conductors are important bioelectronics materials that can potentially be used in a wide variety of biomedical applications, from cardiovascular [15], muscle [16], and nerve tissue engineering [17] to implantable and wearable human-health-monitoring biosensors [18]. Effective synthesis and stabilization methods for AgNPs with narrow size distributions must be developed to mitigate these challenges. Silver’s higher reactivity than gold necessitates careful control over the synthesis process. Additionally, AgNPs interact non-specifically with bacterial membranes and other cellular structures, leading to structural alterations that increase membrane permeability and trigger mitochondrial dysfunction. Consequently, the controlled release of AgNPs is essential to maintain their antimicrobial effectiveness.

Hydrogels are three-dimensional, cross-linked networks of hydrophilic polymers that are water-insoluble yet capable of absorbing and retaining large amounts of water or other guest molecules within their porous structure. This feature, which mimics natural tissues, renders them highly biocompatible and has garnered significant interest in biomaterial applications over the past several decades [19]. Polyhydroxyethylmethacrylate (PHEMA) was the first synthetic hydrogel developed in 1936 by researchers at DuPont [20]. Later, Wichterle and Lim recognized its potential for contact lens applications [21], sparking widespread interest among biomaterial scientists [22].

This initial success spurred the development of “smart hydrogels”, which can respond to external stimuli, such as changes in pH, temperature, ionic strength, magnetic fields, and electric fields [23,24]. The functionalization and structural properties of these hydrogels can be tailored through the use of various co-monomers, polymers, and crosslinkers during synthesis [25]. Functional groups, such as -SO_3_H, -COOH, -NH_2_, -CONH_2_, and -OH are typically present in the monomers used to develop these hydrogels [26,27]. NIPA and AAm are widely used monomers for hydrogel synthesis, offering thermoresponsive and mechanical properties, respectively. AAc and AMPS act as functional comonomers, enhancing hydrogel properties through pH responsiveness and improved ionic conductivity. NCHGs are a specific class of hydrogels incorporating nanomaterials (e.g., metals, nonmetals, metal oxides, and polymeric moieties) into their hydrated polymeric networks. These materials exhibit superior performance compared to conventional hydrogels [28]. By controlling interactions between nanoparticles and polymer chains, it is possible to engineer a wide range of physical, chemical, and biological properties, facilitating applications in catalysis, electronics, biosensing, drug delivery, nanomedicine, and environmental remediation [29]. To promote eco-friendly solutions, various synthesis methods have been devised to produce silver nanocomposites by integrating metal ions into the porous hydrogel matrix. Silver nanoparticle-based wound dressings have received clinical approval; however, reports of dermal toxicity remain a concern [30]. Therefore, combining a hydrogel system with controlled AgNPs would be a better choice for antibacterial activity and treating wounds in the form as such. Hydrogel matrices are appropriate for AgNPs rather than conventional nanocomposite, polymers, bio-macromolecules, dendrimers, liquid crystals, latex particles, etc.

The present study focuses on fabricating electro-conductive, bioactive nanocomposite hydrogels utilizing homogeneous dispersions of AgNPs into polymer networks through three distinct routes. The release of AgNPs from the hydrogel matrix can be externally triggered by adjusting the temperature and pH of the surrounding medium. Additionally, increasing the concentration of AgNPs improves the hydrogels’ conductivity. A simple free radical polymerization method is employed to synthesize these electro-conductive and bioactive nanocomposite hydrogels. This process involves using monomers and co-monomers in the presence of N,N-methylenebisacrylamide (BIS) as a crosslinker and potassium persulfate (KPS) as an initiator. The antibacterial activities of the hydrogels are thoroughly investigated, and their conductivity is measured using impedance spectroscopy. Moreover, the wound-healing properties of these hydrogels are assessed in rat models, providing insights into their potential applications in biomedical fields.

## 2. Results and Discussion

**Scheme 1:**
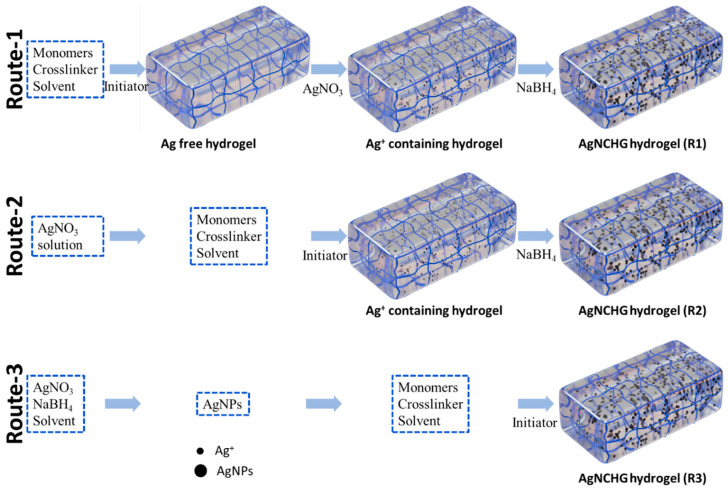
Synthesis of AgNCHGs via three different routes.

Three distinct routes were developed for synthesizing various AgNCHGs. In Route-1, the pure hydrogel was first immersed in an AgNO_3_ solution, allowing Ag^+^ ions to diffuse into the porous hydrogel structure. The electronegative portions of functional groups (e.g., –COO^−^, –NH_2_, –SO_3_^−^) within the monomers bound with the Ag^+^ ions, forming Ag^+^-ion-containing hydrogels. Subsequently, these hydrogels were reduced using NaBH_4_, resulting in the formation of AgNPs containing AgNCHG (R1) (Scheme 1). Approximately 87 mg of AgNO_3_ salt was required to synthesize 1 g of AgNCHG (R1) through this method. The amount of AgNPs within the hydrogel network depends on the close interaction of Ag^+^ ions with the electronegative hydrophilic parts of the monomers.

**Figure 1:**
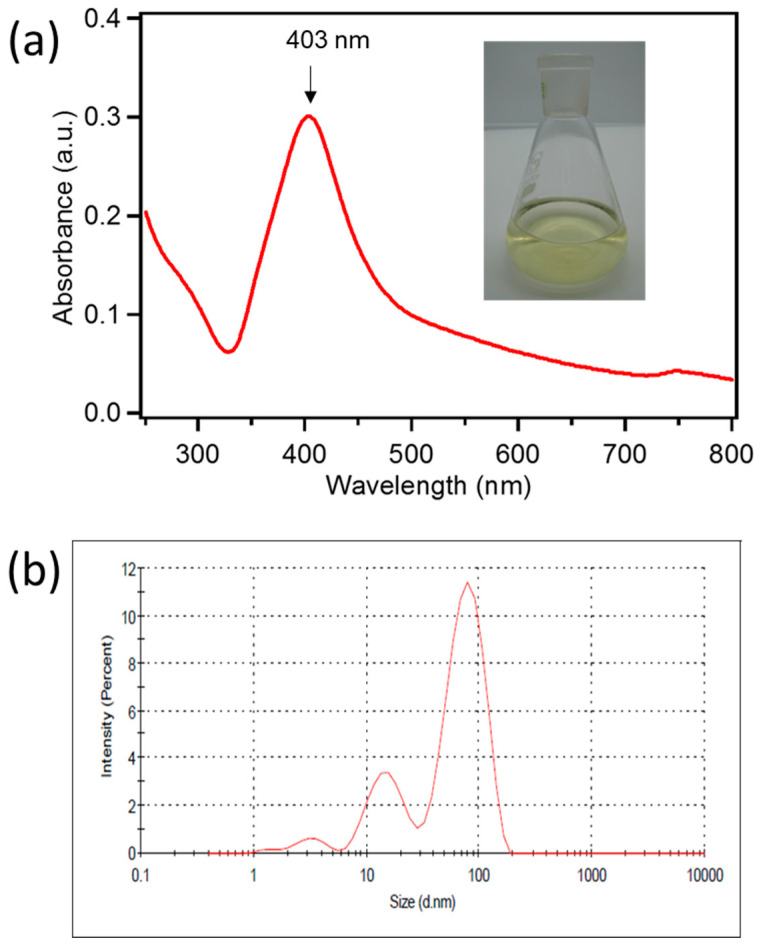
(**a**) UV–Visible spectra of AgNPs colloidal solution and (**b**) hydrodynamic radius of AgNPs from dynamic light scattering.

In Route-2, all gel precursors (except the initiator and accelerator) were mixed with the AgNO_3_ solution, ensuring that the Ag^+^ ions were in close contact with the electronegative portions of the monomers. This process allowed a greater uptake of Ag^+^ ions compared to Route-1. Consequently, after reducing the Ag^+^-ion-containing hydrogel with NaBH_4_, a higher concentration of AgNPs was present in the AgNCHGs (R2) compared to AgNCHGs (R1). Route-2 required only 28 mg of AgNO_3_ salt to synthesize 1 g of AgNCHG (R2), a significantly lower amount than in Route-1. In Route-3, a 40 mM AgNP colloidal solution was prepared and used as the solvent for hydrogel synthesis. This method resulted in the synthesized AgNCHG (R3) containing the lowest amount of AgNPs among the three routes. During the AgNP synthesis, AgNO_3_ served as the source of Ag^+^ ions, which were reduced to neutral silver atoms (Ag^0^) by electrons from reducing agents. Through nucleation and growth, these Ag atoms aggregated to form AgNPs. The distinct surface plasmon resonance peak at 403 nm (Figure 1a) confirmed the formation of spherical AgNPs with a particle size ranging from 25 to 70 nm.

The dynamic light scattering (DLS) results indicated that the average hydrodynamic radius of the synthesized nanoparticles was approximately 42 nm (Figure 1b). The polydispersity index (PDI) of the AgNPs was 0.160, and the sharp peak observed in the analysis confirmed that the nanoparticles were not widely dispersed. The zeta potential of the AgNPs was measured to be −14.2 mV, which contributes to their moderate stability in water.

**Figure 2:**
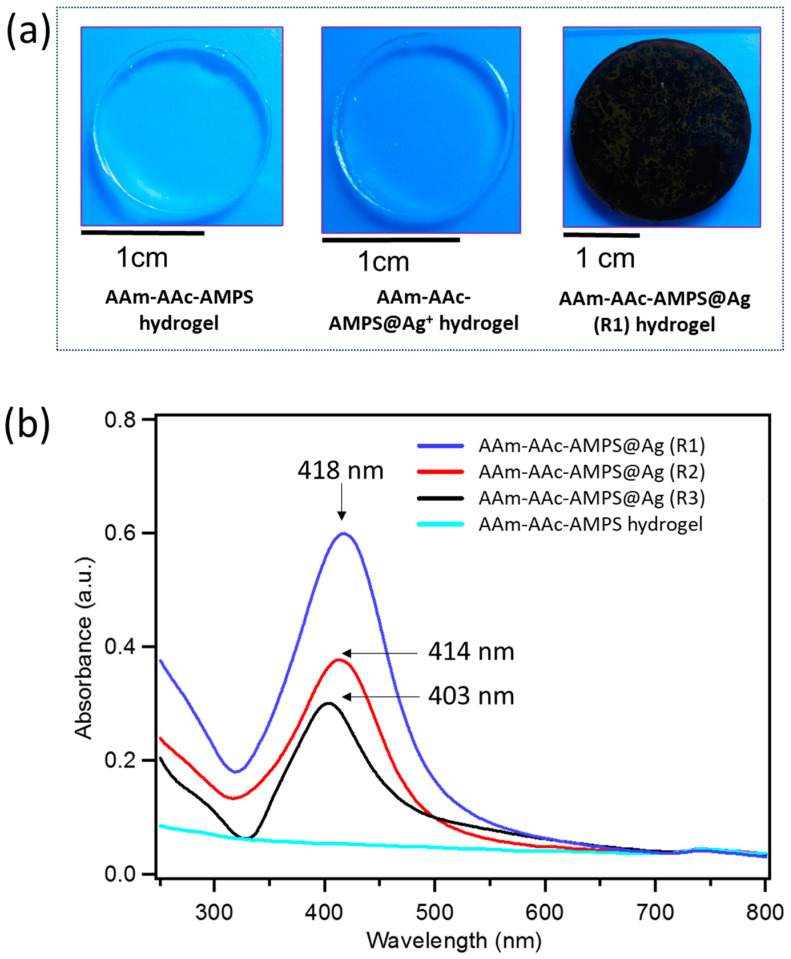
(**a**) Photographs of AAm-AAc-AMPS, AAm-AAc-AMPS@Ag^+^, and AAm-AAc-AMPS@Ag (R1) hydrogels and (**b**) UV–Visible spectra of Ag-free AAm-AAc-AMPS and (AAm-AAc-AMPS)@Ag hydrogels, synthesis via Route-1, Route-2, and Route-3.

All fabricated hydrogels, both Ag-free and Ag^+^-containing, exhibited high transparency and uniformity. However, as the concentration of AgNPs increased in the AgNCHGs, the hydrogels progressively darkened (Tables S1–S3). In all AgNCHGs, AgNPs exist inside the polymer network with excellent stability. Figure 2b presents the UV–Visible spectra of four samples: Ag-free AAm-AAc-AMPS hydrogels and three AgNCHGs (AAm-AAc-AMPS@Ag) synthesized via three different routes. The single plasmon resonance peaks for the Route-1, Route-2, and Route-3 AAm-AAc-AMPS@Ag hydrogels appeared at 418 nm, 414 nm, and 403 nm, respectively, indicating the successful formation of spherical AgNPs [6]. Similar peaks were detected for AAm-AAc@Ag and NIPAm-AAc@Ag AgNCHGs, while no such peaks were present in the Ag-free hydrogel. These findings were further validated by FE-SEM and XRD analyses.

**Figure 3:**
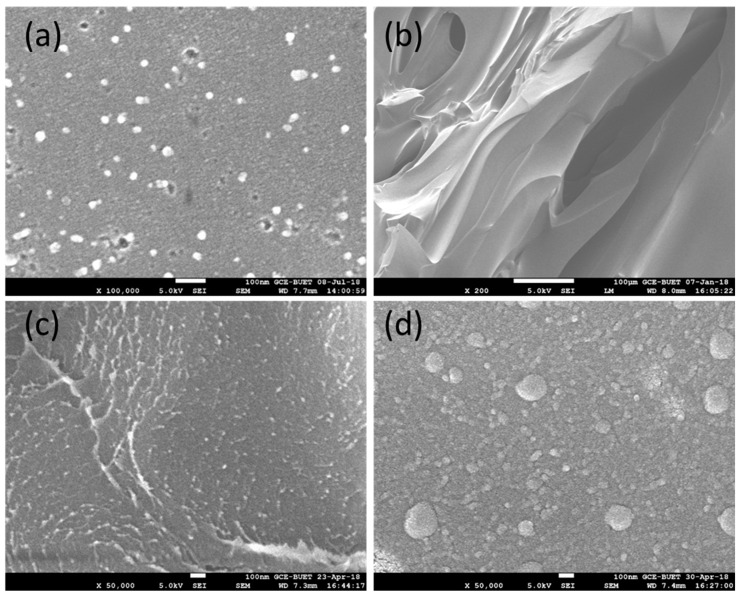
FE-SEM images of (**a**) AgNPs, (**b**) Ag-free hydrogel, (**c**) AAm-AAc-AMPS@Ag (R1), and (**d**) AAm-AAc-AMPS@Ag (R2).

The surface morphology of the synthesized AgNPs, Ag-free hydrogels, and AgNP-containing AgNCHGs was analyzed using FE-SEM. The AgNPs were observed to be spherical, with sizes ranging from 17 nm to 35 nm and an average particle size of approximately 25 nm (Figure 3a). The FE-SEM images of the Ag-free AAm-AAc-AMPS hydrogel (Figure 3b) revealed a smooth surface with a slightly microporous structure.

In contrast, distinct surface morphologies were observed for AgNP-incorporated hydrogels. Figure 3c shows the FE-SEM image of AAm-AAc-AMPS@Ag (R1) AgNCHGs, while Figure 3d shows the FE-SEM image of AAm-AAc-AMPS@Ag (R2) AgNCHGs. These images confirm that the AgNPs were homogeneously dispersed within the polymeric hydrogel network and predominantly spherical. In Route-1, the particle sizes ranged from 10 nm to 30 nm, with an average size of approximately 18 nm. In Route-2, both the quantity and size of the AgNPs were higher, as the hydrogel absorbed a greater amount of Ag^+^ ions. After reduction with NaBH_4_, the resulting nanoparticles were slightly larger, ranging from 25 nm to 45 nm, with an average size of about 35 nm. Similar surface morphologies and size distributions were observed for AAm-AAc@Ag and NIPAm-AAc@Ag AgNCHGs.

**Figure 4:**
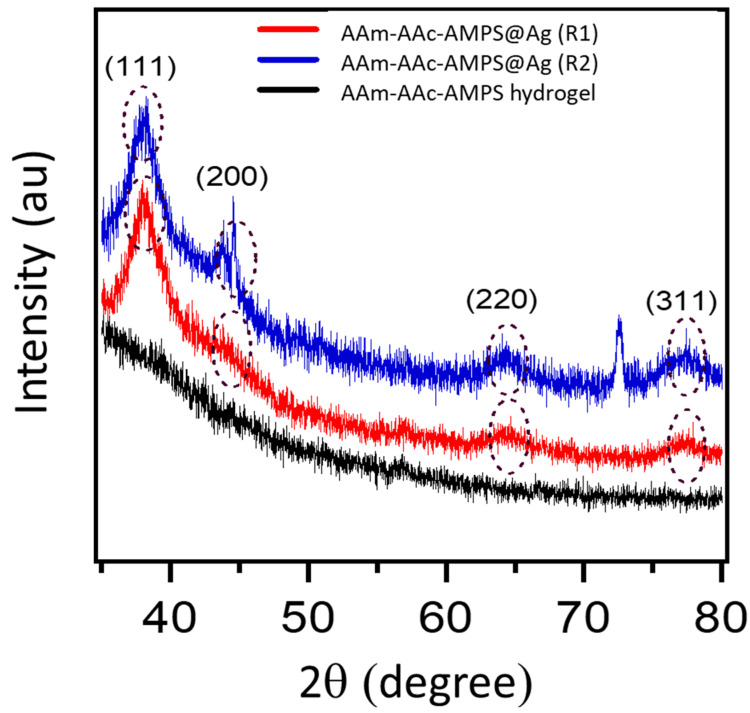
XRD spectra of Ag-free AAm-AAc-AMPS hydrogel, AAm-AAc-AMPS@Ag (R1), and AAm-AAc-AMPS@Ag (R2) AgNCHGs.

The crystalline properties, size, and phase of the samples were characterized using XRD with a Philips Expert PRO X-ray diffractometer. The diffractogram was obtained using Cu-Kα radiation (λ = 0.15406 nm) within the 35° to 80° 2θ range. The XRD patterns for the synthesized Ag-free hydrogel and AAm-AAc-AMPS@Ag AgNCHGs are presented in Figure 4.

Figure 4 presents the XRD peaks of AAm-AAc-AMPS@Ag (R1) hydrogels, observed at 38°, 43.84°, 64.38°, and 77.64°, corresponding to reflections from the (111), (200), (220), and (311) lattice planes, respectively. Similarly, for AAm-AAc-AMPS@Ag (R2) hydrogels, peaks were observed at 37.89°, 43.88°, 64.5°, and 77.59°, corresponding to the same lattice planes. These diffraction patterns confirm the face-centered cubic (FCC) phase of the Ag crystals, consistent with the JCPDS file no. 04-0783. Additionally, the presence of these peaks verifies that silver ions were successfully reduced to their metallic (Ag^0^) state within the hydrogel network [31,32]. In contrast, the XRD spectra of the Ag-free AAm-AAc-AMPS hydrogel did not exhibit any peaks related to AgNPs. The average crystallite sizes of the AgNPs were calculated using the Debye–Scherrer equation [33], further confirming the structural properties of the synthesized nanocomposites.

(1) $$D=\frac{K\lambda }{\beta cos\theta }$$

where D is the average crystallite size, λ = 0.15406 nm is the wavelength of Cu Kα radiation, β is the full width at half maximum (FWHM) of the diffraction peak on the 2θ scale, and θ is the Bragg angle. The D of Ag in the AAm-AAc-AMPS@Ag (R1) hydrogel was calculated to be 9 nm, while in the AAm-AAc-AMPS@Ag (R2) hydrogel, it was 10 nm. These values are consistent with the FE-SEM images, further supporting the observed morphology and size distribution of the AgNPs within the hydrogels. Hydrogels prepared using other routes with different monomers and comonomers also exhibited the same crystalline nature of AgNCHGs, as confirmed by their XRD patterns (Figure S1).

**Figure 5:**
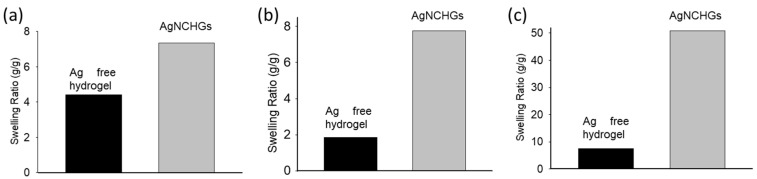
Swelling ratio of Ag-free hydrogel and AgNCHGs hydrogels, (**a**) AAm-AAc-AMPS and AAm-AAc-AMPS@Ag hydrogels, (**b**) AAm-AAc and AAm-AAc@Ag hydrogels, and (**c**) NIPAm-AAc and NIPAm-AAc@Ag hydrogels.

Figure 5 illustrates the swelling behavior of Ag-free and AgNCHGs, including AAm-AAc-AMPS@Ag, AAm-AAc@Ag, and NIPAm-AAc@Ag. The swelling ratio is a key property of hydrogels and nanocomposite hydrogels. The most polar and hydrophilic groups are initially hydrated upon water absorption, forming “primary bound water”. As hydration progresses, the network expands, exposing hydrophobic groups that interact with water molecules, forming “secondary bound water”. Together, primary and secondary bound water are referred to as “total bound water”. Once water interacts with the hydrophilic and hydrophobic regions of the hydrogel, osmotic forces within the network chains drive further water absorption. This continues until equilibrium is reached between the retraction force and the force of infinite dilution, resulting in an equilibrium swelling level. Any water absorbed beyond the total bound water is termed “free water” or “bulk water” [34]. In the case of AgNCHGs, the hydrophilic portions of the polymer chains become more ionic due to the presence of AgNPs, in comparison to Ag^+^-containing or bulk hydrogels. This ionic nature induces stronger repulsive forces among polymer chains with similar charges, causing the polymer network to expand and achieve a higher swelling ratio. Additionally, the primary water interacts with the ionic regions of the polymer network, shielding existing charges. The presence of varying amounts of AgNPs and their increased surface charges further moderates the expansion of the hydrogel networks. As a result, AgNP-containing AgNCHGs exhibit a higher swelling ratio compared to Ag-free hydrogels.

Swelling behavior is significantly influenced by the properties of the polymers and environmental factors such as the pH and temperature of the surrounding medium. When a polymer network interacts with a solution, it swells due to the thermodynamic compatibility between the polymer chains and the solution. This compatibility leads to interactions between the polymer and the solvent molecules, causing the polymer network to expand and absorb solvent, thus increasing its volume. The extent of swelling is further governed by the specific interactions between functional groups in the polymer and the solution. Figure S2 illustrates the swelling ratios of three different types of AgNCHGs across a temperature range of 10 to 50 °C. The AAm-AAc-AMPS@Ag and AAm-AAc@Ag hydrogels show negligible changes in swelling ratio with temperature. In contrast, the NIPAm-AAc@Ag hydrogel exhibits temperature-dependent swelling behavior, attributed to the thermo-responsive monomer NIPAm in the hydrogel network [35]. The value of pH significantly affects the swelling behavior of AgNCHGs containing AAc and AMPS monomers (Figure S3). The Figure S3 reveals a slight, gradual increase in the swelling ratio under acidic conditions from pH 2.0 to 4.0, followed by a sharp transition from pH 4.0 to 7.0. This behavior is attributed to the pKa of the carboxylic acid-containing copolymer AAc, which is approximately 4.5. At pH > 4, the carboxyl groups in the hydrogels dissociate, resulting in increased repulsion among the ionic polar portions of the polymer chains. This enhances the osmotic pressure within the hydrogel, leading to higher swelling. In the pH range of 7.0 to 9.0, the system becomes increasingly basic, and the concentration of basic cations in the surrounding solution rises. As a result, the mobile ion concentration in the outer solution increases, causing a sharp transition in swelling behavior within this range. However, the swelling ratio decreases above pH 7.0 because the dissociation of –COOH groups and the increase in mobile ions lead to a reduction in osmotic pressure, thereby limiting further swelling. From the swelling study, it can be concluded that the swelling ratio of AgNCHGs can be effectively regulated by varying the types and concentrations of monomers. Furthermore, the swelling ratio can be influenced and triggered by environmental stimuli such as pH and temperature.

**Figure 6:**
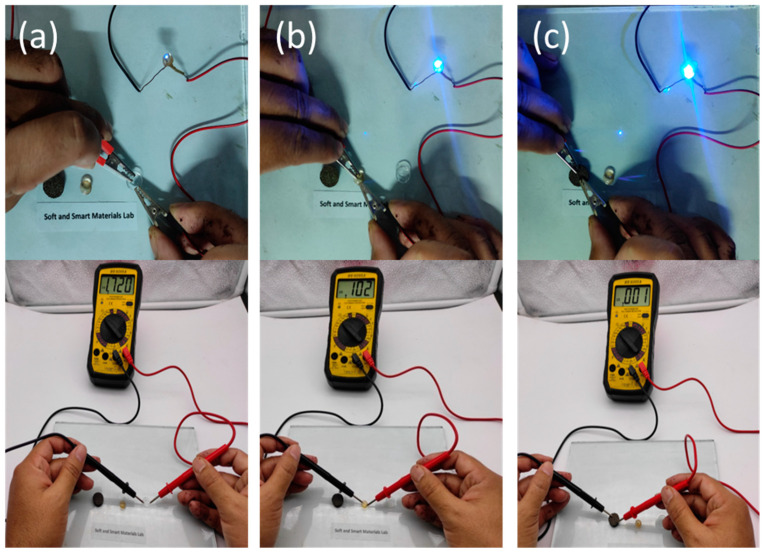
Qualitative and quantitative conductivity measurements of (**a**) Ag-free AAm-AAc hydrogel, (**b**) AAm-AAc@Ag^+^ hydrogel, and (**c**) AAm-AAc@Ag (R1) AgNCHGs.

The conductivity of AAm-AAc hydrogel, AAm-AAc@1.5 M Ag^+^ hydrogel, and AAm-AAc@1.5 M Ag hydrogel was systematically analyzed using an electronic LED circuit and a multimeter (Figure 6). The glowing intensity of the LED bulb served as a qualitative measure of electrical conduction, while the multimeter provided quantitative resistance data. For the AAm-AAc hydrogel, a moderate LED glow and relatively high resistance were observed, indicating baseline conductivity. Incorporating 1.5 M AgNO_3_ into the hydrogel significantly enhanced ionic conductivity, as evidenced by a brighter LED glow and reduced resistance, attributed to the presence of Ag^+^ ions. The highest conductivity was achieved with the AAm-AAc@1.5 M Ag AgNCHG, where the LED glowed brightest, and resistance reached its lowest level, reflecting the efficient conductive pathways provided by the AgNPs. These findings demonstrate a progressive enhancement in conductivity with the addition of AgNO_3_ and AgNPs, showcasing their effectiveness in improving the hydrogel’s electrical performance.

**Figure 7:**
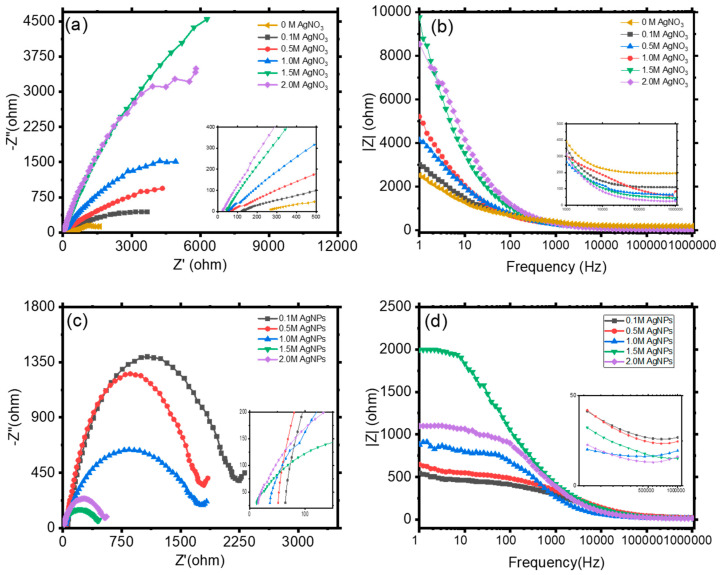
(**a**) Nyquist plots featuring resistance of AAm-AAc hydrogel, AAm-AAc @0.1 M Ag^+^, AAm-AAc @0.5 M Ag^+^, AAm-AAc @1 M Ag^+^, AAm-AAc @1.5 M Ag^+^, and AAm-AAc @2 M Ag^+^ hydrogel. (**b**) The Bode plot featuring resistance of AAm-AAc hydrogel, AAm-AAc @0.1 M Ag^+^, AAm-AAc @0.5 M Ag^+^, AAm-AAc @1 M Ag^+^, AAm-AAc @1.5 M Ag^+^, and AAm-AAc @2 M Ag^+^ hydrogel. (**c**) Nyquist plots featuring resistance of AAm-AAc @0.1 M Ag AgNCHG, AAm-AAc @0.5 M Ag AgNCHG, AAm-AAc @1 M Ag AgNCHG, AAm-AAc @1.5 M Ag AgNCHG, and AAm-AAc @2 M Ag AgNCHG. (**d**) The Bode plot featuring resistance of AAm-AAc @0.1 M Ag AgNCHG, AAm-AAc @0.5 M Ag AgNCHG, AAm-AAc @1 M Ag AgNCHG, AAm-AAc @1.5 M Ag AgNCHG, and AAm-AAc @2 M Ag AgNCHG.

The EIS was conducted on AAm-AAc hydrogels with varying concentrations of AgNO_3_ (0.1 M, 0.5 M, 1 M, 1.5 M, and 2 M) to evaluate their conductivity, as illustrated in the Nyquist plot (Figure 7a). All samples exhibited a straight line parallel to the y-axis, indicative of capacitive behavior [36]. As the AgNO_3_ concentration increased up to 1.5 M, the equivalent series resistance (ESR) decreased, and the length of the straight line along the y-axis increased, reflecting enhanced ionic conductivity and capacitive performance. However, at 2 M AgNO_3_, the ESR increased, and the length of the straight line decreased compared to the 1.5 M AgNO_3_ hydrogel, indicating a decline in ionic transport and capacitive efficiency beyond the optimal AgNO_3_ concentration. The conductivity of these hydrogels was further analyzed through EIS, as shown in the Bode plot (Figure 7b). For the AAm-AAc hydrogel and hydrogels with AgNO_3_ concentrations up to 1.5 M, the impedance (Z) increased at lower frequencies and decreased at higher frequencies, consistent with typical capacitive behavior and improved ionic conductivity with increasing AgNO_3_ concentrations [37]. In contrast, the hydrogel containing 2 M AgNO_3_ displayed lower Z at lower frequencies and higher Z at higher frequencies compared to the 1.5 M AgNO_3_ hydrogel, indicating suboptimal ionic transport dynamics at higher AgNO_3_ concentrations.

The EIS was performed on AAm-AAc hydrogels with varying concentrations of AgNPs (0.1 M, 0.5 M, 1 M, 1.5 M, and 2 M). The Nyquist plots (Figure 7c) revealed a semicircle at mid-frequency, with their characteristics changing according to the AgNP concentration. For hydrogels containing up to 1.5 M AgNPs, the ESR and semicircle area progressively decreased, indicating improved charge transfer and ionic conductivity [38]. However, at 2 M AgNPs, both the ESR and semicircle area increased relative to the 1.5 M AgNP hydrogel, suggesting a decline in conductivity and charge transfer efficiency at higher AgNP concentrations. The Bode plot (Figure 7d) further illustrates the EIS results for AAm-AAc@Ag AgNCHG with varying AgNP concentrations. For hydrogels with AgNP concentrations up to 1.5 M, the impedance (Z) increased at lower frequencies and decreased at higher frequencies, signifying enhanced ionic conductivity and capacitive behavior with increasing AgNPs content. In contrast, for the hydrogel containing 2 M AgNPs, Z was lower at lower frequencies and higher at higher frequencies than the 1.5 M AgNP hydrogel, indicating disrupted ionic transport and charge transfer dynamics at higher AgNP concentrations [39]. These results suggest the potential of these AgNCHGs to energy, sensor, and stretchable electronic sector.

Recent studies have explored whether AgNPs are effective against a broad spectrum of bacterial and fungal species, including antibiotic-resistant strains [40]. Many AgNP-based antibacterial materials have been developed to combat these strains. While AgNPs are potent antibacterial agents, their direct application on injured animal skin is not feasible without supporting materials. AgNCHGs, on the other hand, are biocompatible and closely resemble natural tissues in their physical properties due to their high water content and soft, rubbery consistency. This makes hydrogels ideal for treating infections in burns, traumatic wounds, and as wound dressings. They are even suitable for dry wounds as they do not require additional wound fluids to form gels [34,41,42]. In this study, the antibacterial properties of the synthesized AgNCHGs were evaluated using the disk diffusion method against *E. coli*. SketchAndCalc area calculator software (Release date: 6/21/2018 iCalc, Bristol, UK) was used to measure irregular zone of inhibition (ZOI). The Ag-free hydrogel exhibited no antibacterial activity, whereas all AAm-AAc-AMPS@Ag AgNCHGs prepared via three different routes demonstrated notable antibacterial effects against *E. coli* (Table 1 and Figures S4–S10). Among them, the AAm-AAc-AMPS@Ag (R2), which contained the highest amount of AgNPs, showed the largest ZOI. In contrast, the AAm-AAc-AMPS@Ag (R3), with the lowest AgNP content, exhibited the smallest ZOI. The order of ZOI for the three routes was Route-2 > Route-1 > Route-3. This variation in ZOI can be attributed to the differing amounts of AgNPs present in the hydrogels. Similarly, Ag-free AAm-AAc hydrogel and three AgNCHG samples (AAm-AAc@Ag) synthesized via different routes were tested. The Ag-free hydrogel showed no antibacterial activity, while all AgNCHG samples displayed good antibacterial performance and followed the same ZOI order like AAm-AAc-AMPS@Ag AgNCHGs. The antibacterial tests of Ag-free NIPA-AAc hydrogel and NIPA-AAc@Ag (R1) AgNCHGs were also tested. NIPA-AAc@Ag (R1) AgNCHG exhibited antibacterial activity, whereas the Ag-free hydrogel did not. The ZOI depends on the release rate of AgNPs from the hydrogel, which is influenced by the interaction between the hydrogel matrix and AgNPs. Hydrogels with highly electronegative species, such as carboxylic groups, exhibit stronger interactions with AgNPs, leading to a slower release rate. Consequently, AAm-AAc@Ag hydrogels, which contain a higher proportion of carboxylic acid monomers, release AgNPs more slowly than AAm-AAc-AMPS@Ag hydrogels. As a result, AAm-AAc-AMPS@Ag hydrogels show higher antibacterial activity. The overall ZOI order among the three types of silver nanocomposite hydrogels was AAm-AAc-AMPS@Ag > NIPA-AAc@Ag > AAm-AAc@Ag. This demonstrates the superior antibacterial performance of AAm-AAc-AMPS@Ag hydrogels compared to AAm-AAc@Ag hydrogels.

The demand for new antifungal drugs is growing, but a lack of novel therapeutic targets hinders progress. Here, AgNCHG was tested against wheat blast, a devastating wheat disease caused by the fungus Magnaporthe oryzae. The results, shown in Figure S11, indicate that AgNCHG demonstrated modest activity against Magnaporthe oryzae.

**Figure 8:**
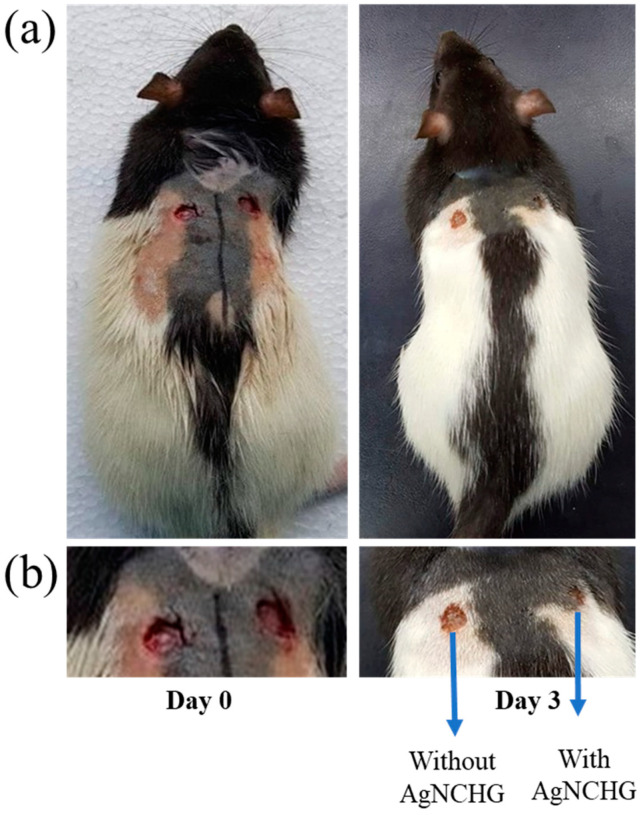
Photographs of wound recovered with and without using AgNCHG after 3 days: (**a**) general appearance; (**b**) magnified appearance.

Wounds created on Long Evans male rats (weight ~220 g) treated with AgNCHG demonstrated significant recovery, with the wound area nearly healed within three days (Figure 8). In comparison, wounds left untreated with AgNCHG exhibited a slower recovery and showed small healed areas during the same timeframe (Figure 8).

## 3. Conclusions

This study successfully developed AgNCHGs through three distinct synthesis routes, each demonstrating considerable biocidal activity, including antibacterial, antifungal, and wound-healing properties. The antibacterial efficacy of the hydrogels was primarily influenced by the concentration of AgNPs within the polymer network. Hydrogels with higher AgNP concentrations exhibited superior antibacterial activity, as the AgNPs played a vital role in generating the observed ZOI. Among the three synthesis routes, Route-2 was identified as the most efficient, being simpler, less time-consuming, and cost-effective. Furthermore, AgNCHGs synthesized via Route-2 featured a homogeneous distribution of AgNPs within the hydrogel matrix and achieved higher AgNP concentrations, thereby enhancing their biocidal activity. The electrical properties of the AAm-AAc hydrogel, which lacked AgNPs, demonstrated the lowest conductivity at 1.1432 × 10^−3^ S cm^−1^. However, incorporating AgNPs significantly enhanced the hydrogel’s conductivity, with an observed increase of 2841.36%. This dramatic improvement highlights the dual role of AgNPs as conductive and biocidal agents. Among the AgNCHGs, AAm-AAc@Ag hydrogels exhibited higher conductivity compared to AAm-AAc@Ag^+^ hydrogels. The enhanced conductivity in AAm-AAc@Ag hydrogels can be attributed to the metallic nature of AgNPs, which act as conductive wires within the network. In contrast, AAm-AAc@Ag^+^ hydrogels relied solely on ionic conduction from free ions, resulting in comparatively lower conductivity. Additionally, the increasing concentration of AgNPs within the hydrogel led to a progressive improvement in conductivity. This observation aligns with percolation theory, which posits that conductivity improves as the concentration of conductive particles surpasses a critical threshold, forming a continuous conductive network. The multifunctional capabilities of AgNCHGs pave the way for future medical, energy, and environmental applications, particularly in wound healing, infection control, antimicrobial coatings, supercapacitor, stretchable electronics, etc.

## 4. Materials and Methods

### 4.1. Chemicals and Reagents

The chemicals and reagents used in this study were purchased from Sigma-Aldrich, Burlington, VT, USA, [including acrylamide (AAm), acrylic acid (AAc), 2-acrylamido-2-methylpropane sulfonic acid (AMPS), N,N-methylenebisacrylamide (BIS), potassium persulfate (KPS), ethanol (CH_3_CH_2_OH), silver nitrate (AgNO_3_), and sodium borohydride (NaBH_4_)]. Oxoid™ Azithromycin Antimicrobial Susceptibility discs were purchased from Thermo Fisher Scientific, Waltham, MA, USA. Liquid nitrogen is used to freeze dry hydrogel before morphological analysis. All chemicals and reagents were of analytical grade and used without additional purification. Deionized water was used as the solvent for preparing the majority of the solutions.

### 4.2. Methods of Preparation of AgNCHGs

#### 4.2.1. Fabrication of AgNCHG of Poly(Acrylamide-Co-Acrylic Acid-Co-2-Acrylamido-2-Methylpropane Sulfonic Acid) Hydrogel, i.e., AAm-AAc-AMPS@Ag Hydrogel

Route-1: A mixture of AAm, AAc, AMPS, BIS, TEMED, and H_2_O was placed in a vial and stirred continuously until a clear solution was obtained (Table S1). A KPS (initiator) solution was prepared in water in a separate container. Both solutions were degassed by bubbling nitrogen gas for 30 min to remove dissolved oxygen. The KPS solution was then added to the first solution under an ice bath. The polymerization was carried out at room temperature for 24 h. The resulting hydrogel was soaked in distilled water for three days to remove unreacted reagents, with the water being replaced every six hours. The purified hydrogel was then immersed in a 6 mM AgNO_3_ solution for 24 h to form an Ag^+^-loaded hydrogel, designated as AAm-AAc-AMPS@Ag^+^. This hydrogel was immediately transferred to a 12 mM NaBH_4_ solution and left for 12 h, forming AAm-AAc-AMPS@Ag (R1) AgNCHG.

Route-2: A solution containing AgNO_3_ and all gel precursors (excluding KPS and TEMED) was prepared in a vial and stirred continuously until it became clear. Separately, a KPS solution was prepared in water. Both solutions were degassed by bubbling nitrogen gas for 30 min to remove dissolved oxygen. The KPS solution was then added to the gel precursor solution in an ice bath. Polymerization was allowed to proceed at room temperature for 24 h. The resulting hydrogel was rinsed in distilled water for three days to remove unreacted chemicals. The Ag^+^-loaded hydrogel, designated as AAm-AAc-AMPS@Ag^+^, was subsequently transferred into a NaBH_4_ solution and left for 12 h, yielding AAm-AAc-AMPS@Ag (R2) AgNCHG. This route eliminates the need for an accelerator during gelation.

Route-3: AgNPs were first synthesized using an established method [6]. Briefly, 50 mL of 2 mM NaBH_4_ aqueous solution was prepared and cooled under constant stirring at 400 rpm for 30 min. Subsequently, 2 mL of 1 mM AgNO_3_ solution was added dropwise at one drop per second. Once all the AgNO_3_ was added, stirring was stopped, and the reaction was allowed to proceed at room temperature [6]. The synthesized aqueous AgNP dispersion was then mixed with all gel precursors (excluding KPS) under continuous stirring to ensure a well-dispersed solution. A KPS solution was prepared in water in a separate container. Both solutions were then transferred into separate test tubes and degassed by bubbling nitrogen gas for 30 min to remove dissolved oxygen. The KPS solution was subsequently added to the gel precursor mixture under an ice bath. Polymerization was carried out at room temperature for 24 h, forming AAm-AAc-AMPS@Ag (R3) AgNCHG. The obtained hydrogel was immersed in distilled water for three days to remove unreacted reagents, with the water being replaced periodically.

#### 4.2.2. Fabrication of AgNCHG of Poly(Acrylamide-Co-Acrylic Acid) Hydrogel, i.e., AAm-AAc@Ag Hydrogel

AAm-AAc@Ag hydrogels were also fabricated via the above-mentioned routes (Table S2).

#### 4.2.3. Fabrication of AgNCHG of Poly(N-Isopropylacrylamide-Co-Acrylic Acid) Hydrogel, i.e., NIPAm-AAc@Ag Hydrogel

The NIPAm-AAc@Ag hydrogel was synthesized only via Route-1, as other methods proved unsuitable for the fabrication of AgNCHGs. All gel precursors, including NIPAm, AAc, BIS, and AIBN, were dissolved in DMSO and stirred continuously until a clear solution was obtained (Table S3). The resulting solution was transferred to a test tube, and nitrogen gas was bubbled through it for 30 min to remove dissolved oxygen. Following nitrogen bubbling, free radical polymerization was performed at 60 °C for 24 h in an oven. The formed hydrogel was immersed in distilled water for seven days to remove unreacted chemicals, with the water being replaced every six hours. After thorough washing, the hydrogel was immersed in a 6 mM AgNO_3_ solution for 24 h to produce an Ag^+^-loaded hydrogel. This hydrogel was immediately transferred to a 12 mM NaBH_4_ solution and kept for 12 h, forming NIPAm-AAc@Ag (R1) AgNCHG.

### 4.3. Sample Characterizations

#### 4.3.1. Spectroscopic Analysis

The UV–Visible spectral analysis was carried out using a double-beam UV–Visible spectrophotometer (Shimadzu-1800, Kyoto, Japan) within a wavelength range of 400–800 nm. The spectra of dried AgNCHG hydrogel samples were recorded in their well-dispersed aqueous states. The dispersion was prepared by stirring 3 g of dried AgNCHG in 10 mL of distilled water continuously for 48 h. The prepared sample was placed in the sample cuvette, while the reference cuvette was filled with the corresponding solvent. All measurements were performed at room temperature, 30 °C (±2 °C). The structural and functional group analyses of the samples were performed using a Fourier transform infrared (FTIR) spectrophotometer (SHIMADZU FTIR-8400, Kyoto, Japan).

#### 4.3.2. Dynamic Light Scattering (DLS)

A DLS analysis was performed using a Malvern Nano ZS instrument (Malvern, UK) at 25 °C with a detection angle of 90°. This technique was primarily used to determine the hydrodynamic radius and zeta potential of AgNPs in aqueous media.

#### 4.3.3. Field Emission Scanning Electron Microscopy (FE-SEM)

The surface morphology of the synthesized AgNPs, Ag-free hydrogel, and AgNCHG was analyzed using a FE-SEM (JSM-7600F, Tokyo, Japan). For the AgNPs sample, a small amount was drop-cast onto a smooth glass slide and dried in an oven. Hydrogel samples, on the other hand, were frozen using liquid nitrogen and subsequently dried using a freeze dryer (Heto FD3, Allerod, Denmark). Once completely dried, the samples were attached to a conductive carbon strip and mounted in the microscope chamber. To ensure surface conductivity, a thin layer of platinum, only a few nanometers thick, was sputtered onto the sample. The microscope operated at an accelerating voltage of 5.0 kV during the analysis.

#### 4.3.4. X-Ray Diffraction (XRD)

The crystallinity and phase identification of the samples were assessed using an XRD (Philips Expert Pro, Amsterdam, The Netherlands). AgNCHGs were analyzed in their powdered form using X-ray diffraction patterns. The powder samples were placed into a square aluminum sample holder (40 mm × 40 mm) with a 1 mm deep rectangular cavity (20 mm × 15 mm). The samples were pressed against an optically smooth glass plate to ensure an even surface. The top surface of the sample was aligned flush with the plane of the sample holder. The prepared sample holder was then positioned in the diffractometer for analysis.

#### 4.3.5. Swelling Behaviors of Ag-Free Hydrogel and AgNCHG

The hydrogel was cut into discs with a diameter of 10 mm and a thickness of 2 mm. The initial weight of the hydrogel was recorded before it was immersed in distilled water. After the hydrogel reached its equilibrium swelling state, its weight was measured again. All measurements were conducted at room temperature (30 °C ± 3 °C). The equilibrium swelling ratio (SR) was calculated using the following formula:

SR = W_s_/W_asp_ (2)

Here, W_s_ is the weight of the swollen hydrogel, and W_asp_ is the weight of the as prepared hydrogel. The temperature dependence of the AgNCHGs was observed between 10 °C and 50 °C. A suitable size of AgNCHGs was placed in a cell immersed in distilled water, and the cell’s temperature was controlled using a circulating water bath. The AgNCHGs were maintained at a specific temperature for three hours to facilitate equilibrium swelling, after which the weight was recorded to determine the SR. The pH dependence of the AgNCHGs was observed across a pH range of 2 to 9. The AgNCHGs were immersed in different buffer solutions, with the pH values measured using a pH meter. The hydrogels were left in the pH solution for five hours to reach equilibrium swelling, and the SR was then recorded.

#### 4.3.6. Determination of Antimicrobial Susceptibility by Modified Kirby–Baur Method

The antibacterial activity of the synthesized AgNCHGs against Escherichia coli (*E. coli*) was assessed using the disk diffusion/Kirby–Bauer method [40]. A cotton swab was dipped into a suspension of approximately ~10⁶ colony-forming units (CFU)/mL of a fresh *E. coli* culture and streaked evenly in three directions over the surface of nutrient agar plates to ensure uniform bacterial growth. The plates were then left to dry for 3 to 5 min. Hydrogel samples, along with an Oxoid™ Azithromycin Antimicrobial Susceptibility disc (used as a positive control), were then placed on the surface of the nutrient agar. The concentration of azithromycin in Oxoid™ Azithromycin Antimicrobial Susceptibility disc was 15 µg/disc. The plates were incubated at 37 °C for 24 h. Afterward, the inhibition zones around the disks, where bacterial growth was suppressed, were measured and calculated using the manual antimicrobial susceptibility testing (AST) method.

#### 4.3.7. Wound-Healing Properties of AgNCHG

To evaluate the wound-healing properties of AgNCHG, the hydrogel was applied to skin wounds on Long Evans male rats weighing approximately 220 g. The assessment was conducted over three days. All animal experiments adhered to ethical guidelines and complied with both international and institutional ethical standards. The study was approved by the Institutional Research Ethics Board (IREB) of United International University (UIU) under reference number IREB/2023/037, dated 11 December 2023.

#### 4.3.8. Electrochemical Impedance Analysis (EIA)

The electrochemical experiments were carried out using a two-electrode setup with a computer-controlled electrochemical workstation (CHI 660E, Austin, TX, USA). The electrochemical impedance spectroscopy (EIS) was conducted at room temperature, at the open circuit potential, with an AC amplitude of 10 mV, in the frequency range of 0.01 Hz to 10,000 kHz. According to the size of the total (2 mm to 8.5 mm) of the sample holder of the EIA, Ag-free hydrogel, Ag^+^ containing hydrogel, and AgNCHG hydrogels were cut.

## Figures and Tables

**Table 1:** Values of ZOI of Ag-free hydrogel, AgNCHGs, and antibiotics (Azithromycin) against *E. coli* using agar disc diffusion method.

Samples Code	ZOI (in mm)	Ratios Between the Diameter of ZOI and AgNCHG (in mm) $R=\frac{d_{ZOI}}{d_{AgNCHG}}$
AAm-AAc-AMPS@Ag (R1) hydrogel	19.8	(19.8/6.85) = 2.90
AAm-AAc-AMPS@Ag (R2) hydrogel	25.0	(25.0/8.40) = 2.98
AAm-AAc-AMPS@Ag (R3) hydrogel	12.60	(12.60/8.70) = 1.45
AAm-AAc@Ag (R1) hydrogel	16.0	(16.0/10.0) = 1.6
AAm-AAc@Ag (R2) hydrogel	15.0	(15.0/8.65) = 1.73
AAm-AAc@Ag (R3) hydrogel	13.0	(13.0/10.0) = 1.3
NIPA-AAc@Ag (R1) hydrogel	23.5	(23.5/8.0) = 2.94
Ag-free hydrogel	0	0
Azithromycin	21.75	(21.75/7.0) = 3.10

## Data Availability

The original contributions presented in this study are included in the article/Supplementary Materials. Further inquiries can be directed to the corresponding authors.

## Supplementary Materials

The following supporting information can be downloaded at: https://www.mdpi.com/article/10.3390/gels11020084/s1. Table S1. Recipe of gel precursors to synthesize AAm-AAc-AMPS@Ag hydrogel. Table S2. Recipe of gel precursors to synthesize AAm-AAc@Ag hydrogel. Table S3. Recipe of gel precursors to synthesize NIPAm-AAc@Ag hydrogel. Figure S1. (a) XRD spectra of (a) NIPAm-AAc-AMPS@Ag (R1), AAm-AAc-AMPS@Ag (R1), Am-AAc@Ag (R1), and Am-AAc-AMPS hydrogels. Figure S2. Swelling ratios of three different types of AgNCHGs at different temperature. Figure S3. Swelling ratios of three different types AgNCHGs (a. AAm-AAc-AMPS@Ag; b. AAm-AAc@Ag; c. NIPA-AAc@Ag) hydrogel at different pH levels. Figure S4. Photographs showing the inhibition of growth of bacterial colonies (*E. coli*) in (A) silver-free Hydrogel (B), (C), and (D) are of AAm-AAc-AMPS@Ag (R1), AAm-AAc-AMPS@Ag (R2), and AAm-AAc-AMPS@Ag (R3) AgNCHGs, respectively. Figure S5. Photograph showing the inhibition of growth of bacterial colonies (*E. coli*) in (A) silver-free Hydrogel (B), (C) and (D) AAm-AAc@Ag (R1), AAm-AAc@Ag (R2), and AAm-AAc@Ag (R3) AgNCHGs, respectively. Figure S6. Photograph showing the inhibition of growth of bacterial colonies (*E. coli*) in (A) silver-free Hydrogel and (B) NIPA-AAc@Ag (R1) AgNCHGs. Figure S7. Photograph showing (A) blank Petri dish, (B) growth of bacterial colonies (*E. coli*) and (C) the inhibition of growth of bacterial colonies. Figure S8. Comparison of antibacterial activity among three types of AgNCHGs which are synthesized via Route-1 against *E. coli*. Figure S9. Comparison of antibacterial activity between two types of AgNCHGs which are synthesized via Route-2 against *E. coli*. Figure S10. Comparison of antibacterial activity between two types of AgNCHGs which are synthesized via Route-3 against *E. coli*. Figure S11. Photograph showing of antifungal test of (A) silver-free Hydrogel and (B) AgNCHG against Magnaporthe oryzae.
