# Supplementary material for: Facile Synthesis of Bioactive Silver Nanocomposite Hydrogels with Electro-Conductive and Wound-Healing Properties
Source: Gels. 2025 Jan 22;11(2):84. doi: 10.3390/gels11020084 (PMC11854814; doi:10.3390/gels11020084)
Supplement: Supplementary file 1 [file gels-11-00084-s001.zip › gels-3431657-supplementary.pdf]

Supporting Information

Facile Synthesis of Bioactive Silver Nanocomposite Hydrogels with Electro-Conductive and Wound Healing Properties

Solaiman[1], Tahmina Foyez[2*], Syed Abdul Monim[1], Aminur Rahman[1], Abu Bin Imran[1*]

[1]Department of Chemistry, Bangladesh University of Engineering and Technology, Dhaka-1000, Bangladesh

[2]Department of Pharmacy, United International University, United City, Madani Ave, Dhaka-1212, Bangladesh

Corresponding email: abimran@chem.buet.ac.bd

Table S1. Recipe of gel precursors to synthesize AAm-AAc-AMPS@Ag hydrogel.

**Type**	**AAm (M)**	**AAc (M)**	**AMPS (M)**	**KPS (mM)**	**BIS (mM)**
A	1.90	0.05	0.05	2.96	20.0
B	1.80	0.10	0.10	2.96	20.0
C	1.70	0.15	0.15	2.96	20.0
D	1.60	0.20	0.20	2.96	20.0

Table S2. Recipe of gel precursors to synthesize AAm-AAc@Ag hydrogel.

**Type**	**AAm (M)**	**AAc (M)**	**KPS (mM)**	**BIS (mM)**
A	1.90	0.10	2.96	20.0
B	1.80	0.20	2.96	20.0
C	1.70	0.30	2.96	20.0
D	1.60	0.40	2.96	20.0

Table S3. Recipe of gel precursors to synthesize NIPAm-AAc@Ag hydrogel.

**Type**	**NIPA (M)**	**AAc (M)**	**AIBN (mM)**	**BIS (mM)**
A	1.90	0.10	8.00	20.0
B	1.80	0.20	8.00	20.0
C	1.70	0.30	8.00	20.0
D	1.60	0.40	8.00	20.0

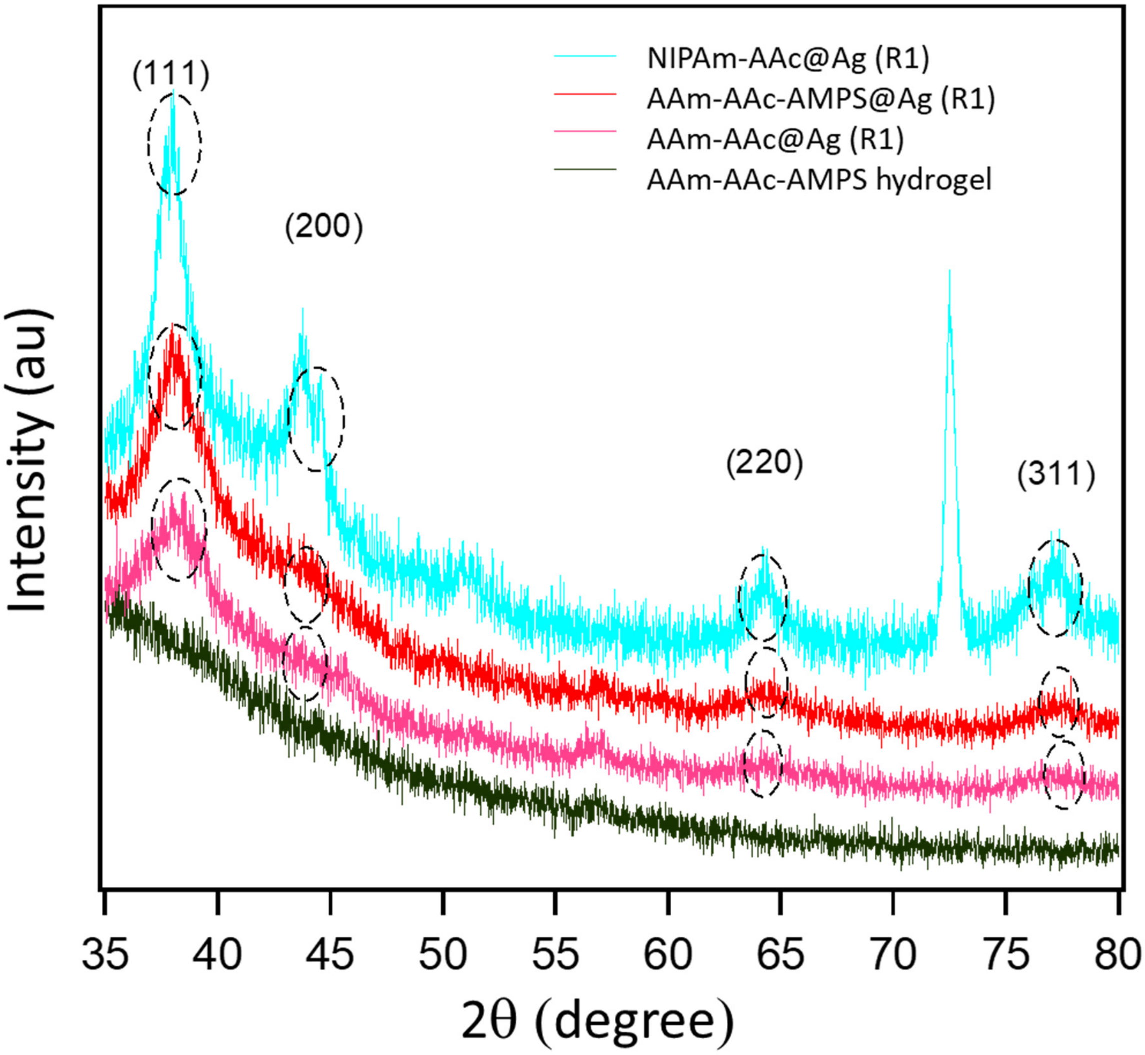

Figure S1. (a) XRD spectra of (a) NIPAm-AAc-AMPS@Ag (R1), AAm-AAc-AMPS@Ag (R1), Am-AAc@Ag (R1) and Am-AAc-AMPS hydrogels.

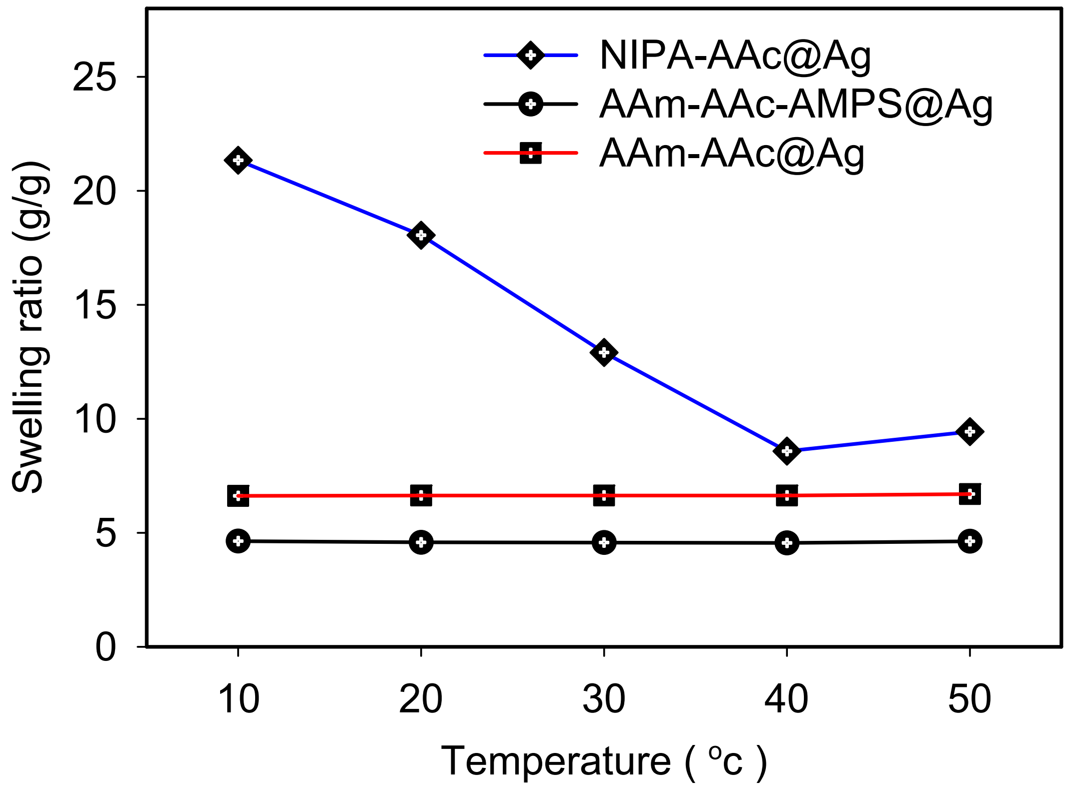

Figure S2. Swelling ratios of three different types of AgNCHGs at different temperature.

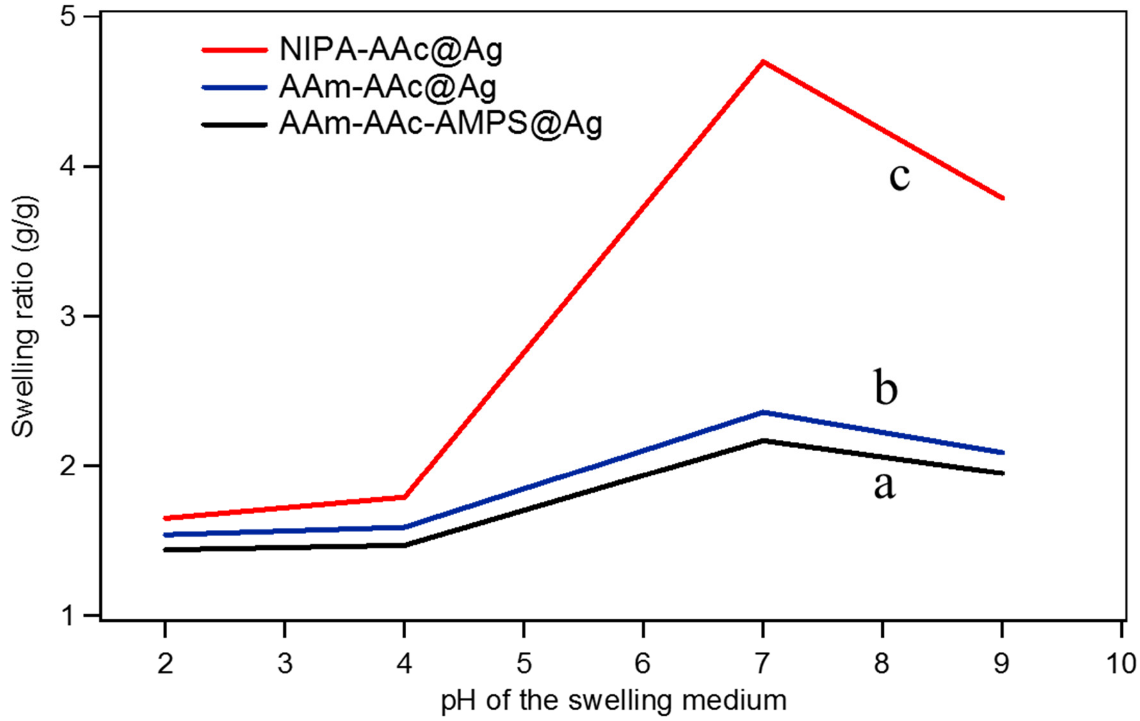

Figure S3. Swelling ratios of three different types AgNCHGs (a. AAm-AAc-AMPS@Ag b. AAm-AAc@Ag c. NIPA-AAc@Ag) hydrogel at different pH.

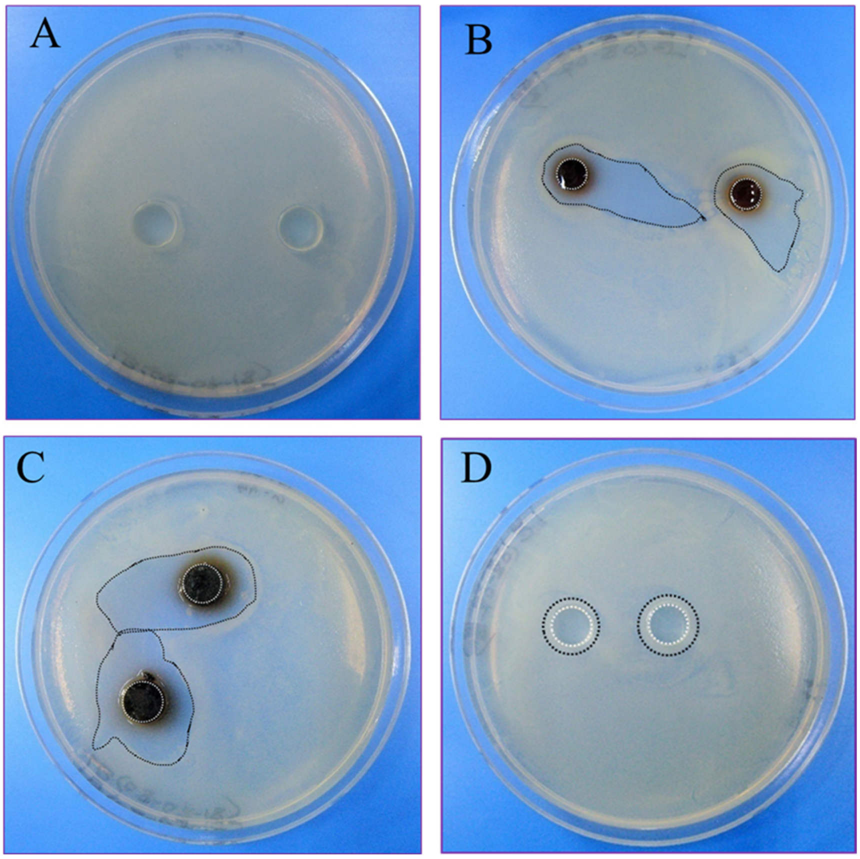

Figure S4. Photographs showing the inhibition of growth of bacterial colonies (*E. coli*) in (A) silver-free Hydrogel (B), (C) and (D) are of AAm-AAc-AMPS@Ag (R1), AAm-AAc-AMPS@Ag (R2), and AAm-AAc-AMPS@Ag (R3) AgNCHGs, respectively.

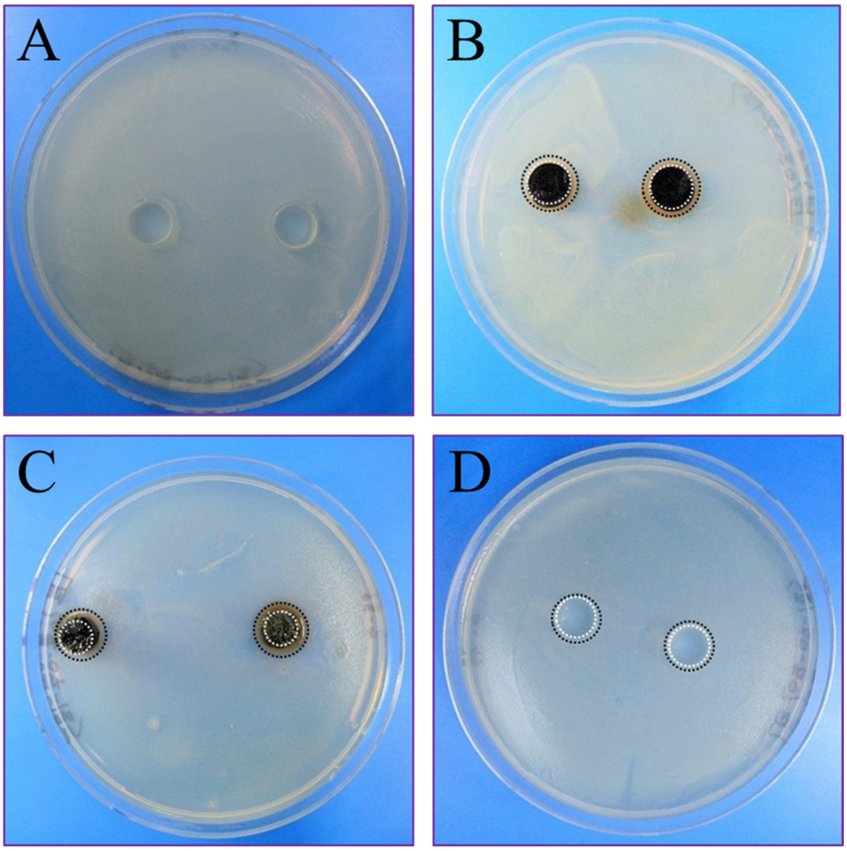

Figure S5. Photograph showing the inhibition of growth of bacterial colonies (*E. coli*) in (A) silver-free Hydrogel (B), (C) and (D) AAm-AAc@Ag (R1), AAm-AAc@Ag (R2), and AAm-AAc@Ag (R3) AgNCHGs, respectively.

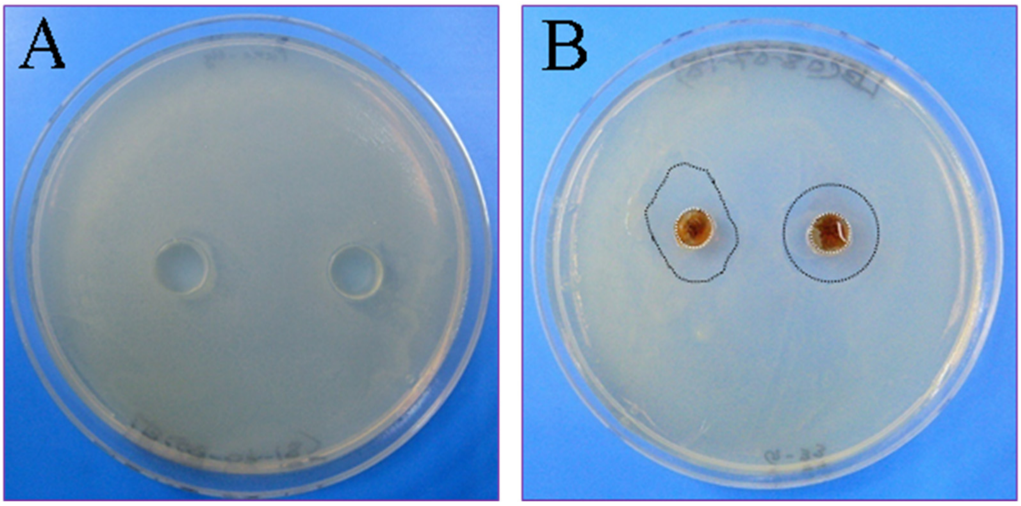

Figure S6. Photograph showing the inhibition of growth of bacterial colonies (*E. coli*) in (A) silver-free Hydrogel and (B) NIPA-AAc@Ag (R1) AgNCHGs.

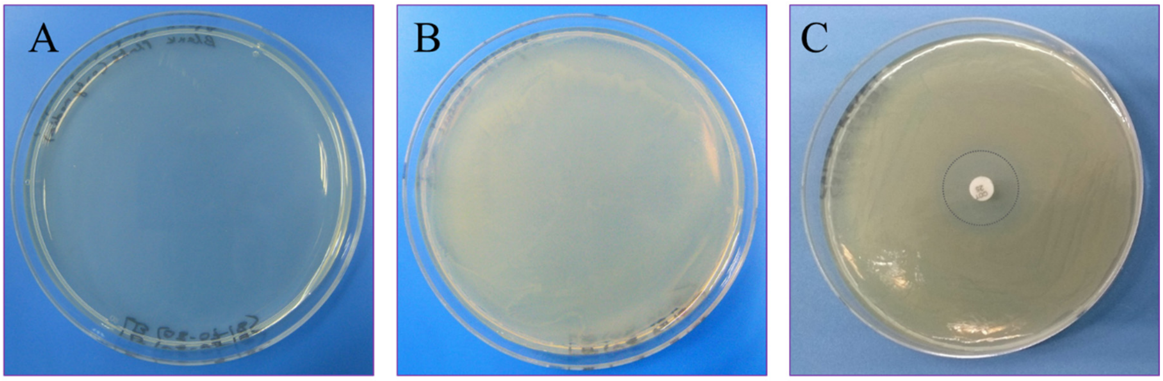

Blank

Negative control (only bacteria growth)

Positive control (Applied azithromycin antibiotics)

Figure S7. Photograph showing (A) blank petri dish, (B) growth of bacterial colonies (*E. coli*) and (C) the inhibition of growth of bacterial colonies.

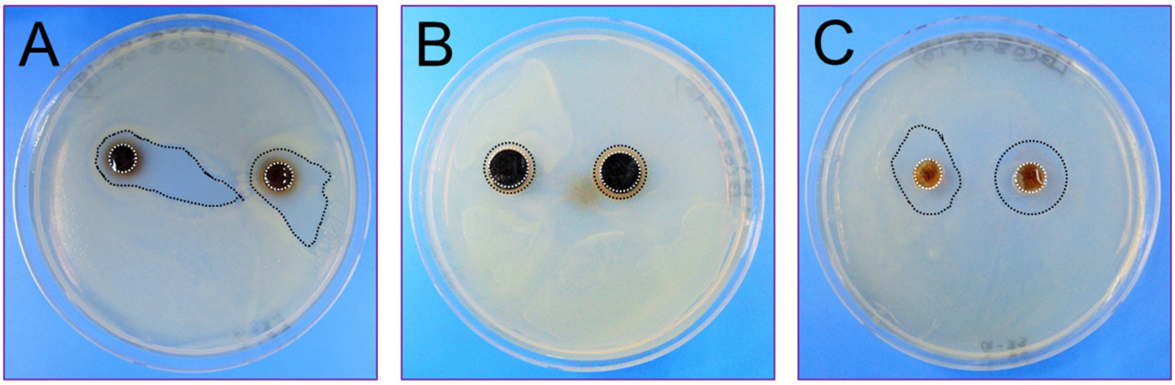

(AAm-AAc-AMPS@Ag) hydrogel

(AAm-AAc@Ag) hydrogel

(NIPA-AAc@Ag) hydrogel

Figure S8. Comparison of antibacterial activity among three types of AgNCHGs which are synthesized via route-1 against *E. coli*.

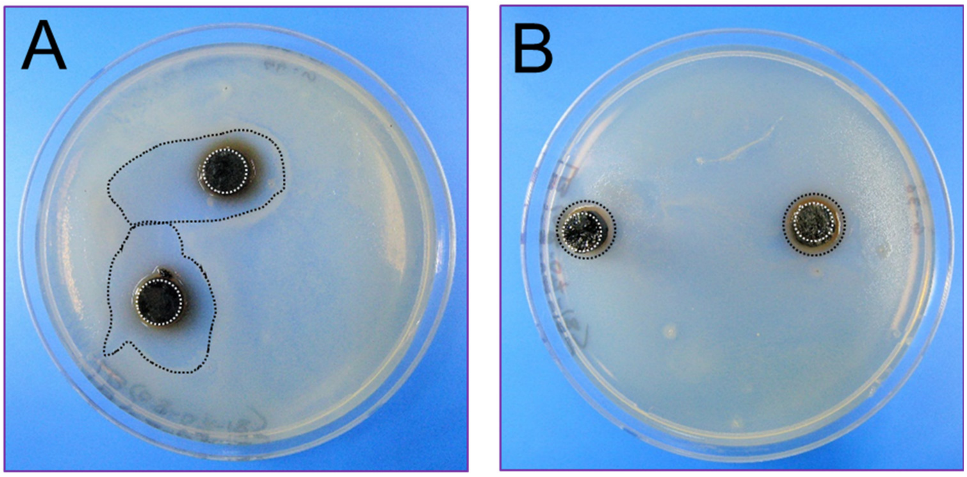

(AAm-AAc-AMPS@Ag) hydrogel

(AAm-AAc@Ag) hydrogel

Figure S9. Comparison of antibacterial activity between two types of AgNCHGs which are synthesized via route-2 against *E. coli*.

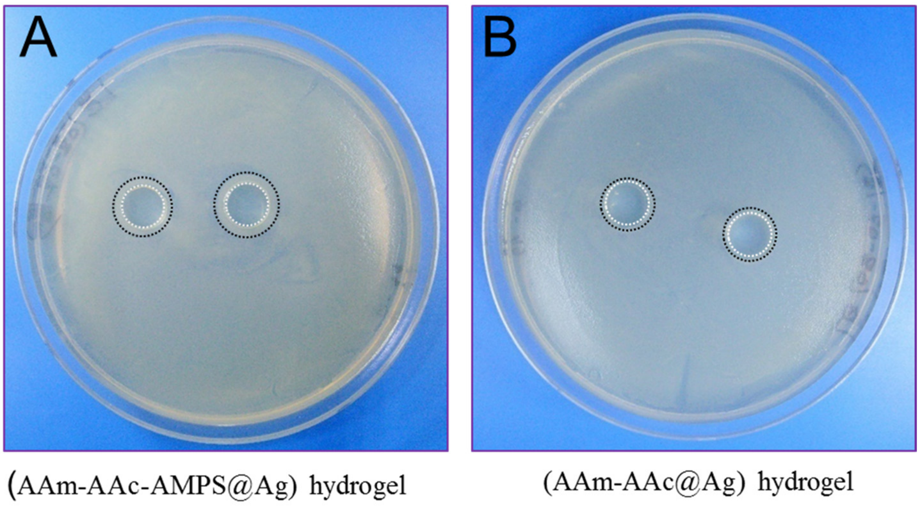

Figure S10. Comparison of antibacterial activity between two types of AgNCHGs which are synthesized via route-3 against *E. coli*.

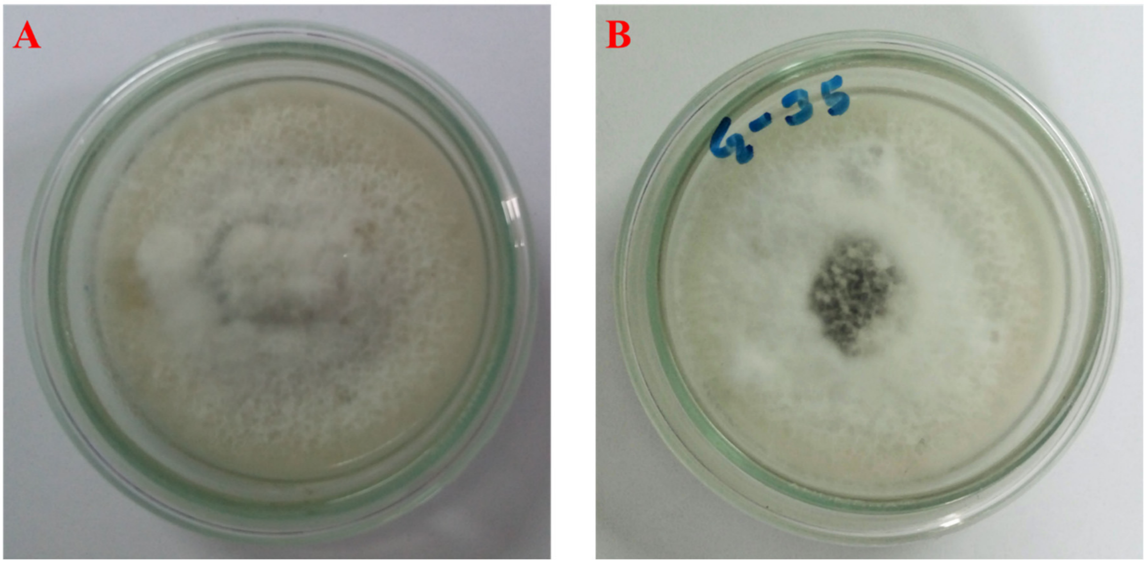

Figure S11. Photograph showing of antifungal test of (A) silver-free Hydrogel and (B) AgNCHG against *Magnaporthe oryzae.*
